# How do Google searches for symptoms, news and unemployment interact during COVID-19? A Lotka–Volterra analysis of google trends data

**DOI:** 10.1007/s11135-020-01089-0

**Published:** 2021-01-30

**Authors:** Chiara Sotis

**Affiliations:** Department of Geography and Environment, London School of Economics and Political Science, London, UK

**Keywords:** Lotka–Volterra, COVID-19, News, Unemployment, Alcohol, Porn

## Abstract

In this paper I exploit Google searches for the topics “symptoms”, “unemployment” and “news” as a proxy for how much attention people pay to the health and economic situation and the amount of news they consume, respectively. I then use an integrable nonautonomous Lotka–Volterra model to study the interactions among these searches in three U.S. States (Mississippi, Nevada and Utah), the District of Columbia and in the U.S. as a whole. I find that the results are very similar in all areas analyzed, and for different specifications of the model. Prior to the pandemic outbreak, the interactions among health searches, unemployment searches and news consumption are very weak, i.e. an increase in searches for one of these topics does not affect the amount of searches for the others. However, from around the beginning of the pandemic these interactions intensify greatly, suggesting that the pandemic has created a tight link between the health and economic situation and the amount of news people consume. I observe that from March 2020 unemployment predates searches for news and for symptoms. Consequently, whenever searches for unemployment increase, all the other searches decrease. Conversely, when searches for any of the other topics considered increase, searches for unemployment also increase. This underscores the importance of mitigating the impact of COVID-19 on unemployment to avoid that this issue swallows all others in the mind of the people.

## Introduction

The COVID-19 pandemic is a threat for public health (Heymann and Shindo 2020) and the economy (Atkeson 2020; Baker 2020; McKibbin and Fernando 2020). As of January 2021, the virus has caused over 389,000 deaths in the U.S. and almost 2 millions worldwide (Worldometers 2020). When COVID-19 first started spreading across the US in early March 2020, States and the Federal Government were forced to limit or shut down a wide array of economic activities to protect public health. This had catastrophic consequences on the economy. The GDP decreased at an annual rate of 31.7% in the second quarter of 2020 (Bureau of Economic Analysis 2020), and 33 million people filed for unemployment between March and May 2020 (The Guardian 2020). These statistics dwarf the impact of the 2007 financial crisis. In turn, this strong economic downturn is likely to have negative repercussions on the healthcare system. As unemployment keeps rising and GDP decreasing, many unemployed Americans become unable to afford the expensive health insurance premia while the States and Federal Governments may not be able to increase public spending to support them. Additionally, despite claims that insurance companies would pay for testing and waive co-payments for treatments, even Americans with a coverage have some reason to worry. Some insurance plans have already set out limitations to their waivers of co-payments (for instance they might waive them only in specific months) and others, such as plans that are not compliant with the Affordable Care Act, may not cover the costs associated with testing at all (CBS 2020). The importance of this cannot be overstated, as recent estimates suggest that total payments to hospitals for treating uninsured patients under the Trump administration policy could range from $13.9 billion to $41.8 billion. At the top end of the range, these payments would consume more than 40% of the $100 billion Fund the Congress created to help hospitals and others respond to the COVID-19 epidemic (KFF 2020). Against this background, it is unsurprising that over 50% of Americans report feeling scared and overwhelmed, and that one of the reactions to the pandemic has been consuming more news (Horowitz Research Center 2020). However, this could trigger a vicious circle because people who consume more news during a time of severe crisis are likely to become even more worried. In fact, Garfin (2020) warn that an increased media exposure during COVID-19 might affect people’s mental health and their level of worry, resulting in damaging consequences to the individuals and society as a whole. The elevated emotional response associated with extreme events, like the pandemic, is also likely to make people more pessimistic, and to heighten anxiety and physical and mental distress.

There are many reasons why news consumption may increase in proximity of the outbreak of a new virus. To begin with, news consumption could increase because of the economic state of country and due to the scientific uncertainty surrounding the virus. State and national governments, along with the WHO and the CDC, are continuously updating guidelines and regulations aimed at containing the virus, so citizens wishing to be informed are likely to read more news and try to understand common symptoms on a regular basis. The economic crisis could also motivate searches for news, as a crisis of this magnitude creates great uncertainty, and people might look at news outlets to help navigating this uncertainty. Additionally, at the beginning of the COVID-19 pandemic the increased volume of searches related to news could be further influenced by the large amount of misinformation circulating on the internet (Imhoff and Lamberty 2020; Pennycook 2020), which led to a 900% increase in fact checks in English language between January and March 2020 (Brennan 2020).

Hence, on the one hand, people are likely to search for more news to better understand the causes and the unfolding of the health and economic crises. On the other hand, causality could go also in the opposite direction, as people who consume more news during a pandemic are likely to become more preoccupied with the health and economic crises and search more terms related to them.

Against this background, it becomes important to find out the relationship between news consumption, symptoms searches and searches related to unemployment. Understanding how some searches can affect citizens to make others can help policymakers communicate more effectively with citizens and structure targeted interventions. For instance, finding that people are extremely preoccupied with unemployment, so much that this search predates all others, can be a signal that absent good welfare protections people are not able to pay sufficient attention to other issues like health. Failing to address citizens’ growing concerns risks jeopardising any new attempt at economic restrictions, e.g. shorter opening times for shops or forbidding gatherings, which may become necessary if a second wave arises. Moreover, the interactions between these common searches can help to identify people’s perceived priorities and their likely reactions to policies.

Many studies have already investigated how COVID-19 affected the level of concern for the health and the economic situation (Mertens 2020; Trueblood 2020), or people’s increased searches for news (Horowitz Research Center 2020) and unemployment filings (Tian and Goetz 2020). However, no study has looked at the simultaneous interactions among these three key consequences of the pandemic. Additionally, addictive behaviors like porn and alcohol consumption can increase exponentially during a time of crisis (Mestre-Bach 2020), can affect people’s mental and physical health (Grubbs et al. 2015; Clay and Parker 2020) and mediate the relationships between searches for news, unemployment and symptoms. Therefore, I carry out the analysis both without including additional mediators in the model, and including them.

I use Google topic searches for the words “symptoms”, “unemployment”, “news” as proxy for the attention that people pay to the health and economic crises and for how much news they consume. Moreover, I use “porn” and “alcoholic drinks” searches as potential mediators. Importantly, the searches considered are not those for the specific terms, but refer to the topics considered and hence encompass all searches related with these topics.

The use and gratification theory suggests possible reasons behind internet usage and which websites people visit (Eighmey and McCord 1998). This theory posits that people actively choose the media to use to satisfy their needs and be gratified (Yi-Cheng et al. 2013). The motivations and needs to satisfy can vary from knowledge enhancement, entertainment and relaxation, social interaction and reward to remuneration (Ko et al. 2005). Among these (Kate Maddox 1998) stresses that obtaining information is of paramount importance. While the scope of this paper is not to identify the primary reason behind the searches made, it is likely that this is even more true in trying times, such as a pandemic. Notwithstanding the many reasons that could drive to these searches, here I focus their interactions. To do so, I use the the nonautonomous Lotka–Volterra model developed in Marasco (2016), and Marasco and Romano (2018a, 2018b) to study how these searches “interact” with each other. I say that there is an interaction between topics when an increase (decrease) in the searches for one has an impact on the number of searches for the other.

There are three advantages in using this nonautonomous Lotka Volterra model to study these interactions. First, since the interaction coefficients are dependent on time, the model allows me to capture changes in the way in which different searches interact and when these changes take place (Dominioni 2019, 2020). This allows me to identify whether the pandemic outbreak affected the strength and type of interactions between the searches Americans make. Second, this model allows me to derive these interactions without having to ask for stated behaviors. Finally, because the solutions of the model are known I do not have to estimate the parameters using expensive numerical methods like genetic algorithms (Romano 2016). Instead, I can derive the competitive roles from the data.

I carry out the analysis with respect to different areas and find that the results are largely identical for all areas included, for all combinations of factors considered and for different specifications of the model. Therefore, they can be considered robust. I find that prior to the COVID-19 outbreak the interactions among health searches, unemployment searches, news and porn (alcoholic drinks) are very weak, but as the pandemic approaches they intensify. These findings show how these factors become tightly interconnected during the pandemic, and therefore that analysing the existing interactions among them is key to understand the consequences of the pandemic. In particular, I find that during the pandemic searches for unemployment predate all others, including searches for symptoms. This suggests a widespread attention for the economic crisis that predates all other factors considered. Conversely, the other searches proceed in mutualism with each other.

## Materials and method

To study the interactions between Google topic searches for unemployment, symptoms, news and porn (alcoholic drinks) I collect weekly data from Google Trends. Google Trends offers information on how popular a given search is on Google. The user can analyze searches for a given “term” or for a “topic”. The former includes only data on searches for a specific term, whereas the latter includes data for all searches related with a given topic, even if searched through different wording. For instance, searches for “unemployment” as a topic would also include searches for “Cares Act” and searches for “news” would include searches for “CNN”. In line with the literature making use of these data, in this paper I use search data for topics. In fact, this kind of data has been used to to forecast short-term economic indicators such as consumer confidence, unemployment and unemployment filings (Varian and Choi 2012; Baker and Fradkin 2017; D'Amuri and Marcucci 2017; Pavlicek and Kristoufek 2015; Tian and Goetz 2020), to understand the magnitude of traffic for porn (Gmeiner 2015) and even to predict the spread of diseases, including COVID-19 (Carneiro and Mylonakis 2009; Ginsberg et al. 2009; Mavragani and Gillas 2020; Nuti et al. 2014; Lu and Reis 2020).

The output of Google Trends data is not the actual number of searches for a specific term or for a topic. Instead, it shows the interest for a given term or topic relative to the other terms or topics included in the analysis. Each data point is normalized by dividing the number of searches for the term by the total number of all searches in that area. Therefore, the algorithm controls for both population differences and the differences in search volume among areas. Google Trends also eliminates repeated searches by a single individual in a short period of time to prevent a single individual from skewing the results. Additionally, data from Google Trends is freely available, highly representative and almost real-time, which makes it a compelling alternative to surveys or social media, which may only capture the opinions of a few people at a specific moment and suffer from self-selection. Moreover, understanding whether searches for some topics affects the share of searches for others through stated behaviors, e.g. in a survey, would require asking respondents directly whether an increase in their searches for a topic, e.g. news, affects their number of searches for others, e.g. unemployment. This can lead to biased results due to focusing bias (the experimenter would need to ask respondents to recall such a relationship) and lack of memory. Additionally, it would raise the costs of research. However, the nature of the data means that individual-level interpretations of the sequencing of searches over time are not warranted and can only be hypothesised.

The areas selected for my search are the United States as a whole, the District of Columbia (DC), Mississippi, Nevada and Utah. These areas were chosen using the following procedure. I input the four search topics considered on Google Trends and restrict the area to United States and time to twelve months, i.e. from May, 5th 2019 to May 5th 2020. After selecting the four topics, it becomes possible to rank the States and U.S. territories by the relative interest in one of the topics compared to the other three topics. Thus, for example, if we rank States for the relative (to the other three topics) interest in unemployment, we see that Nevada comes on top. I select the States that lead for each of the topics. In other words, I select the four “corner” areas based on the idea that the external validity of this study would be higher if the results hold for areas that are at the extremes in term of interest for the topics considered. A side benefit is that this choice of territories allows me to consider two States with a Republic Governor (Mississippi and Utah) and Nevada and DC that have a Democratic Governor and a Democratic major, respectively. Importantly, I choose not to include coronavirus among the topics, as it would dwarf all others. Not only is interest for coronavirus enormous, but the search for coronavirus as a topic would include searches for symptoms and news related to the pandemic, making it impossible to separate searches for the different domains considered here. Hence, to avoid this issue, I do not include the topic coronavirus among the searches considered. To analyze this data I apply the integrable nonautonomous Lotka Volterra model developed in Marasco (2016) and Marasco and Romano (2018a) for which the solutions are known. For an accurate description of the model and the relative formal proof I refer the reader to Marasco (2016). The search shares are written in the form of a logit model. In line with Marasco (2016) the market share $S_{i}(t)$ at time *t* of a search *i* in a given area is the probability of searching topic *i* from all possible $N+1$ topics via the * logit model*, i.e.

1 $$\begin{aligned} S_{i}(t)=\frac{\exp \left( f_{i}\left( t\right) \right) }{\sum \nolimits _{j=0}^{N}\exp \left( f_{j}\left( t\right) \right) },\quad i=0,...,N \end{aligned}$$

where $f_{i}\left( t\right)$ is the *utility function* for the users to search topic *i* at time *t*. Each utility function $f_{i}\left( t\right)$ is defined as a (linear or nonlinear) function of all aspects and attributes impacting the choice between alternative searches. In this case the topic searches are: unemployment ($i=1$), symptoms ($i=2$), news ($i=3$), porn (alcoholic drinks) ($i=4$). Other topic searches ( $i=0$) play the role of the *outside search*. Then, following the microeconomics utility theory (McFadden 1974) Eq. (1) becomes

2 $$\begin{aligned} \left\{ \begin{array}{l} \ S_{i}(t)=\frac{\exp \left( f_{i}\left( t\right) \right) }{1+\sum \nolimits _{j=1}^{N}\exp \left( f_{j}\left( t\right) \right) },\quad i=1,...,N \\ S_{0}(t)=\frac{1}{1+\sum \nolimits _{j=1}^{N}\exp \left( f_{j}\left( t\right) \right) }, \quad \forall t \end{array}\right. \end{aligned}$$

where $S_{0}(t)=1-\sum \nolimits _{i=1}^{N}S_{i}(t)$ at any time *t*. In mathematical terms, the utility functions $f_{i}\left( t\right)$ are defined as a linear or nonlinear combination of suitable time-varying variables $V_{h},h=1,...,M$, and each of them can have a positive or negative influence on the utility functions. Moreover, the market shares of the *i*-th search increases when its utility function $f_{i}\left( t\right)$ increases and decreases when the utility function $f_{j}\left( t\right)$ of any other competitor increases, i.e. when searching one topic is perceived as relatively better (i.e. yields more utility) than searching another. If we assume that all the utility functions $f_{i}\left( t\right) ,i=1,...,N,$ are of class $C^{2}\left( \left[ t_{0},+\infty \right) \right)$ Eqs. (3) are the unique (global) solution of the Cauchy problem

3 $$\begin{aligned} \left\{ \begin{array}{l} \dot{S}_{i}(t)=\dot{f}_{i}\left( t\right) S_{i}(t)\left[ 1-S_{i}(t)\right] -\sum \nolimits _{j=1,j\ne i}^{N}\dot{f}_{j}\left( t\right) S_{j}(t)S_{i}(t),\quad i=1,\cdots ,N, \\ S_{i}\left( t_{0}\right) =\frac{\exp \left( f_{i}\left( t_{0}\right) \right) }{1+\sum \nolimits _{j=1}^{N}\exp \left( f_{j}\left( t_{0}\right) \right) } \end{array} \right. \end{aligned}$$

where the dot denotes the time derivative of a share, $t\in \left[ t_{0},+\infty \right)$ and $S_{0}(t)=1-\sum \nolimits _{i=1}^{N}S_{i}(t).$ The model can be used to study the interaction coefficients between the different searches. In fact, owing to Marasco (2016), Romano (2016) and Marasco and Romano (2018b), Eq. (3)$_{\text {1}}$ belong to the following class of integrable nonautonomous Lotka–Volterra systems

4 $$\begin{aligned} \dot{S}_{i}(t)= \underbrace{g_{i} \left( t \right) S_{i}(t)\left[ 1-S_{i}(t)\right] }_{\text {logistic growth}}- \underbrace{\sum \limits _{j=1,j\ne i}^{N}g_{j}\left( t \right) S_{j}(t)S_{i}(t)}_{\text {interaction with competitors}}, \quad i=1,\cdots ,N, \end{aligned}$$

where $g_{i}\left( t\right) =\dot{f}_{i}\left( t\right)$ for all $t\in \left[ t_{0},+\infty \right) .$ Eq. (4) then describes the interaction between the *i*-th and *j*-th topic search and allows competitive roles to change over time. The evolution of the market share $S_{i}(t)$ of the *i*-th topic search is mathematically determined by two factors: the logistic growth rate function $g_{i}\left( t\right)$ and the competition functions $g_{j}\left( t\right)$ between the *i*-th and *j*-th topic searches. Last, the maximum capacity related to the saturation value of $S_{i}(t),i=1,...N,$ is 1 (i.e., the maximum potential percentage of the market shares). The coefficients $g_{i}\left( t\right)$ in Eq. (4) are constant if and only if the utility functions are linear combinations of the variables $V_{h}$ and these variables depend linearly on time. Then, in most cases the system (4 ) is nonautonomous. How market shares $S_{i}(t)$ vary in time in response to variations of one or more utility functions can be evaluated by Eq. (2). Tables 3, 4, 5, 6 and 7 report the descriptive statistics for each of the variables considered, with the values they take before they are transformed into shares. The descriptive statistics show that people in DC have a relatively high interest for news, whereas people in Mississippi have a relatively high interest for porn. Despite these differences, news is the most searched topic in all territories considered, apart from Mississippi. It is also interesting to note that in Utah people search very little for unemployment, while people in Nevada are the ones searching unemployment the most. To determine the competitive roles between any two variables we look at the signs of the interaction coefficients, i.e. the functions $g_{i}\left( t\right)$ and their intensity by the sum of their absolute values. Hence, according to Table 1 the LV model (4) is able to capture all the possible kinds of competitive interactions.

**Table 1:** The table indicates the possible kinds of interaction between search A and search B, depending on the interaction coefficients of the two searches

Sign of Int. coefficient A	Sign of Int. coefficient B	Interaction	Description
$+$	$+$	Competition	An increase (reduction) in the searches for A negatively (positively) affects the searches for B, and vice versa
−	$+$	Predator–prey	An increase in the value of search A negatively affects the value of search B (predator). An increase in the value of search B positively affects search A (prey)
−	−	Mutualism	An increase (a reduction) in the value of one search increases (reduces) the value of the other
−	0	Commensalism	An increase (decrease) in the value of search A positively (negatively) affects search B. Search A is unaffected by changes in B.
$+$	0	Amensalism	An increase (decrease) in the value of search A negatively (positively) affects index B. Search A is unaffected by changes in B.
0	0	Neutralism	No interaction.

**Fig. 1:**
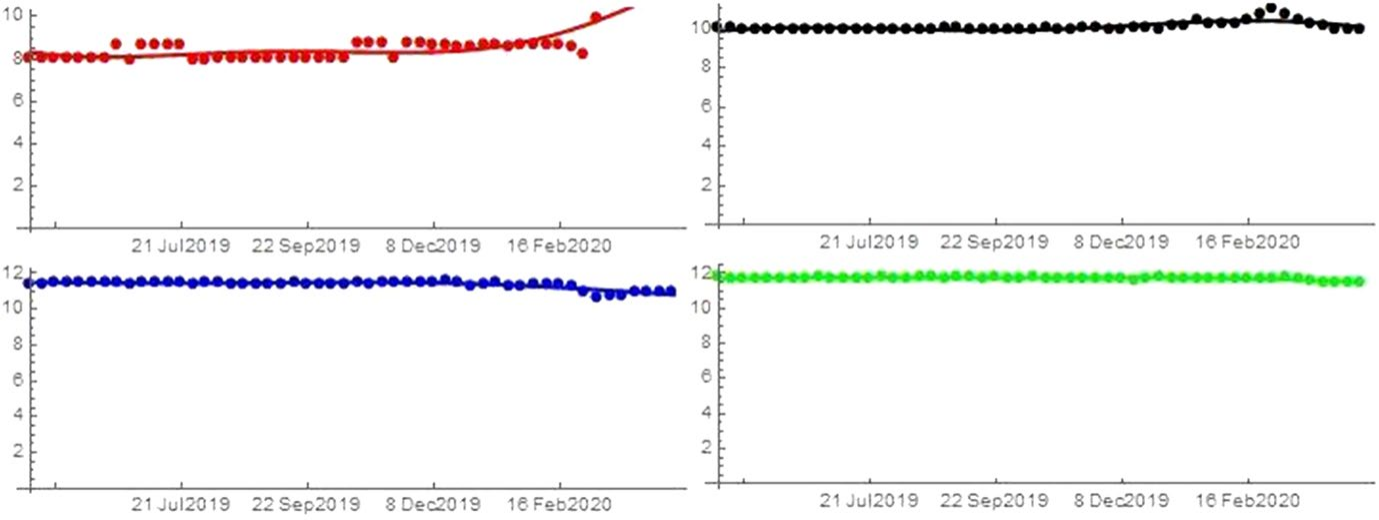
This figure, in order from top to bottom (left to right), shows the fitting for unemployment (red), symptoms (black), porn (blue) and news (green) for United States. (Color figure online)

Importantly, I have data on searches, but not on the utility functions. Consequently, I derive the utility functions from the historical data on searches (transformed in shares) using a fitting procedure. As standard in the literature, I use a Fourier series. The results are similar when using a fourth degree polynomial to fit the data. An example of fitting is reported in Fig. 1. The utility functions are evaluated from $f_{i}\left( t\right)$ starting from the data on market shares as follows

5 $$\begin{aligned} f_{i}\left( t\right) =\ln S_{i}(t)-\ln S_{0}(t)\quad i=1,...,N. \end{aligned}$$

Eq. (5) allows us to determine a discrete set of values for each utility function starting from historical data on market shares.

Therefore, the indirect determination of the analytical form of these functions is obtained by a *fitting procedure* using the *Fourier series of order n*

6 $$\begin{aligned} f_{i}^{F}\left( t\right) =a_{0}+\displaystyle \sum \limits _{r=1}^{n}\left( a_{r}\cos \frac{r\pi t}{\tau }+b_{r}\sin \frac{r\pi t}{\tau }\right) \quad i=1,...,N \end{aligned}$$

where $\tau =T_{1}/2$ if $f_{i}\left( t\right)$ is a periodic function with period $T_{1}$, or $\tau =kT_{1}$ for a suitable $k\ge 1$ otherwise

The findings from different specification are largely the same and do not depend on the variables considered. Therefore, while the analysis below refers to the specification that considers unemployment, symptoms, news and porn, the considerations made apply also to the specifications with: (*i*) unemployment, symptoms, news, and alcoholic drink, and (*ii*) unemployment, symptoms and news.

### Accuracy of the model

I assess the accuracy and reliability of the model using the mean squared error (MSE), a standard measure of error. The MSE of an estimator (in this case the fitting procedure) measures the average of the squares of the errors that is, the average squared difference between the estimated values and the actual value. The MSE is calculated as follows.

7 $$\begin{aligned} MSE=\frac{1}{n}\sum \limits _{i=1}^{n}\left( h_{i}-p_{i}\right) ^{2},\quad \end{aligned}$$

where $h_{i}$ and $p_{i}$ are respectively the historical and predicted values. The measures of error are reported in Table 2.

**Table 2:** This table reports the mean squared error (MSE) of unemployment, symptoms, porn and mews in United States, DC, Mississippi, Nevada and Utah

Area	Unemployment	Symptoms	Porn	News
United States	0.000794175	0.00045305	0.00105343	0.000546758
DC	0.000687643	0.000628962	0.00134754	0.00126896
Mississippi	0.00119979	0.000601171	0.00155282	0.000789139
Nevada	0.00211069	0.000421077	0.00125108	0.00117399
Utah	0.000434824	0.000445824	0.00107998	0.000728572

The MSEs refers to the code that considers unemployment, symptoms porn and news

**Table 3:** Summary statistics for unemployment, symptoms, porn and news for United States

US	Mean	Max	Min	SD
Unemployment	5.596153846	38	1	10.78535611
Symptoms	9.538461538	35	7	5.892705822
Porn	30.98076923	36	28	1.47515881
News	45.15384615	100	36	11.1221112

**Table 4:** Summary statistics for unemployment, symptoms, porn and news for DC

DC	Mean	Max	Min	SD
Unemployment	6.346153846	46	1	11.11593912
Symptoms	9.826923077	40	4	7.508743019
Porn	21.13461538	35	18	3.412944483
News	58.98076923	100	44	9.680160395

**Table 5:** Summary statistics for unemployment, symptoms, porn and news for Mississippi

Mississippi	Mean	Max	Min	SD
Unemployment	6.865384615	54	1	14.51304869
Symptoms	13.73076923	50	9	7.849354625
Porn	53.96153846	66	48	4.63558926
News	50.69230769	100	38	10.39332075

**Table 6:** Summary statistics for unemployment, symptoms, porn and news for Nevada

Nevada	Mean	Max	Min	SD
Unemployment	8.269230769	81	1	16.93439307
Symptoms	6.057692308	50	4	4.054501115
Porn	23.36538462	66	20	1.074688616
News	36.21153846	100	28	11.71346454

**Table 7:** Summary statistics for unemployment, symptoms, porn and news for Utah

Utah	Mean	Max	Min	SD
Unemployment	2.673076923	17	1	4.4312666
Symptoms	9.096153846	32	5	5.520939162
Porn	20.76923077	24	19	1.322733127
News	38.26923077	100	29	12.20111757

## Results

### Results of the LV model

**Fig. 2:**
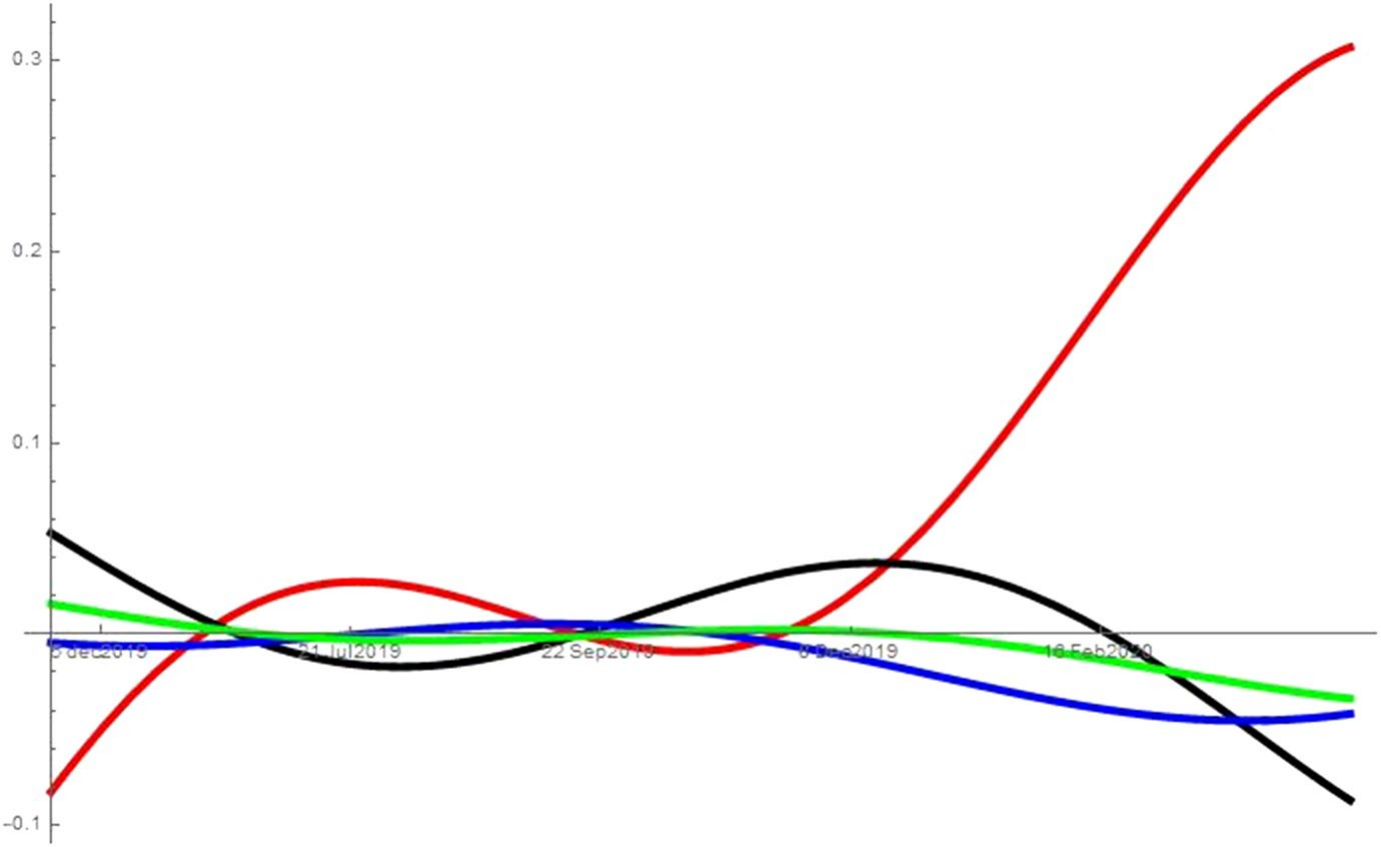
This figure shows the interaction coefficients of unemployment (red), symptoms (black), porn (blue) and news (green) for the United States

**Fig. 3:**
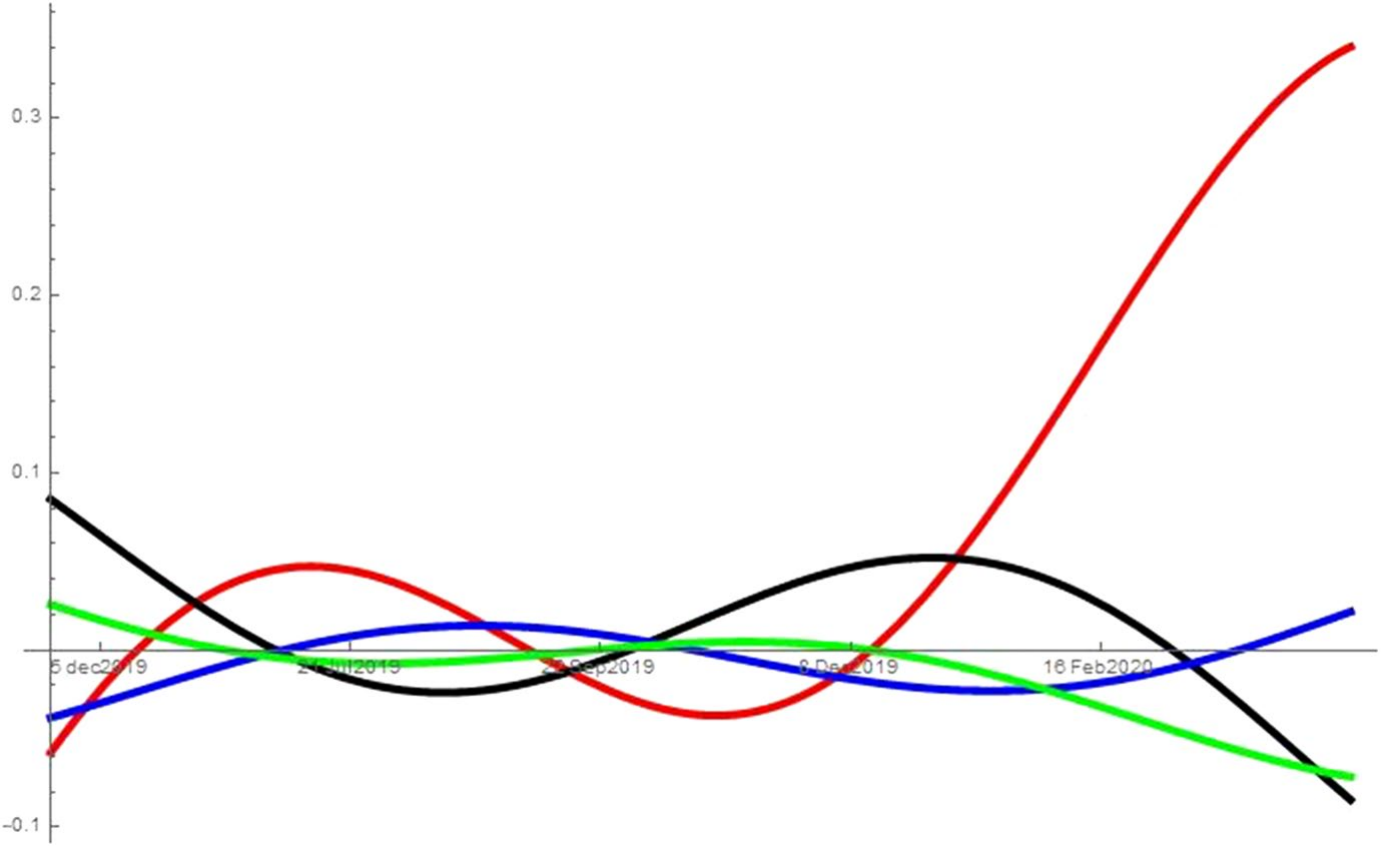
This figure shows the interaction coefficients of unemployment (red), symptoms (black), porn (blue) and news (green) for DC

**Fig. 4:**
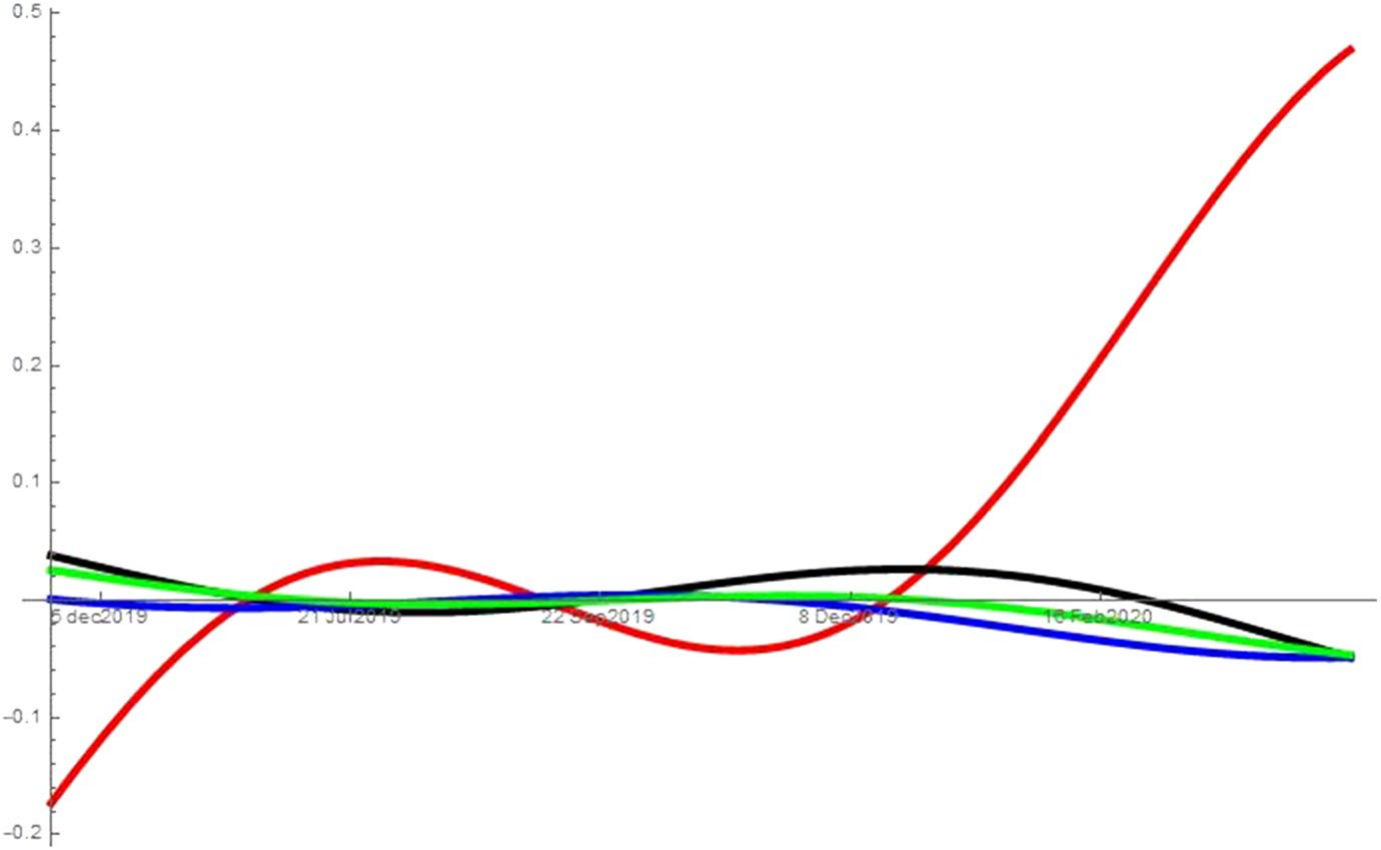
This figure shows the interaction coefficients of unemployment (red), symptoms (black), porn (blue) and news (green) for Mississippi

**Fig. 5:**
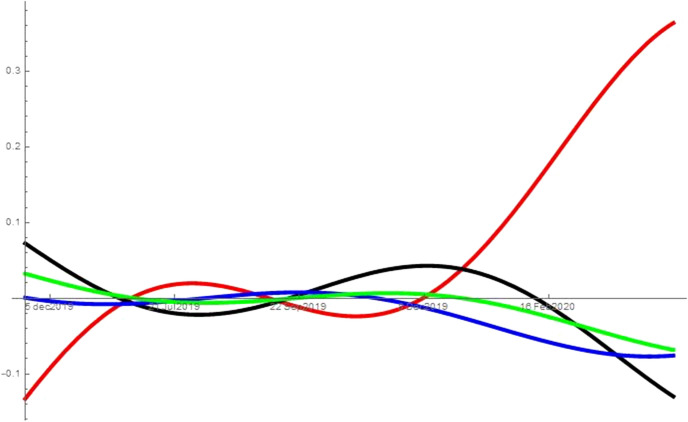
This figure shows the interaction coefficients of unemployment (red), symptoms (black), porn (blue) and news (green) for Nevada

**Fig. 6:**
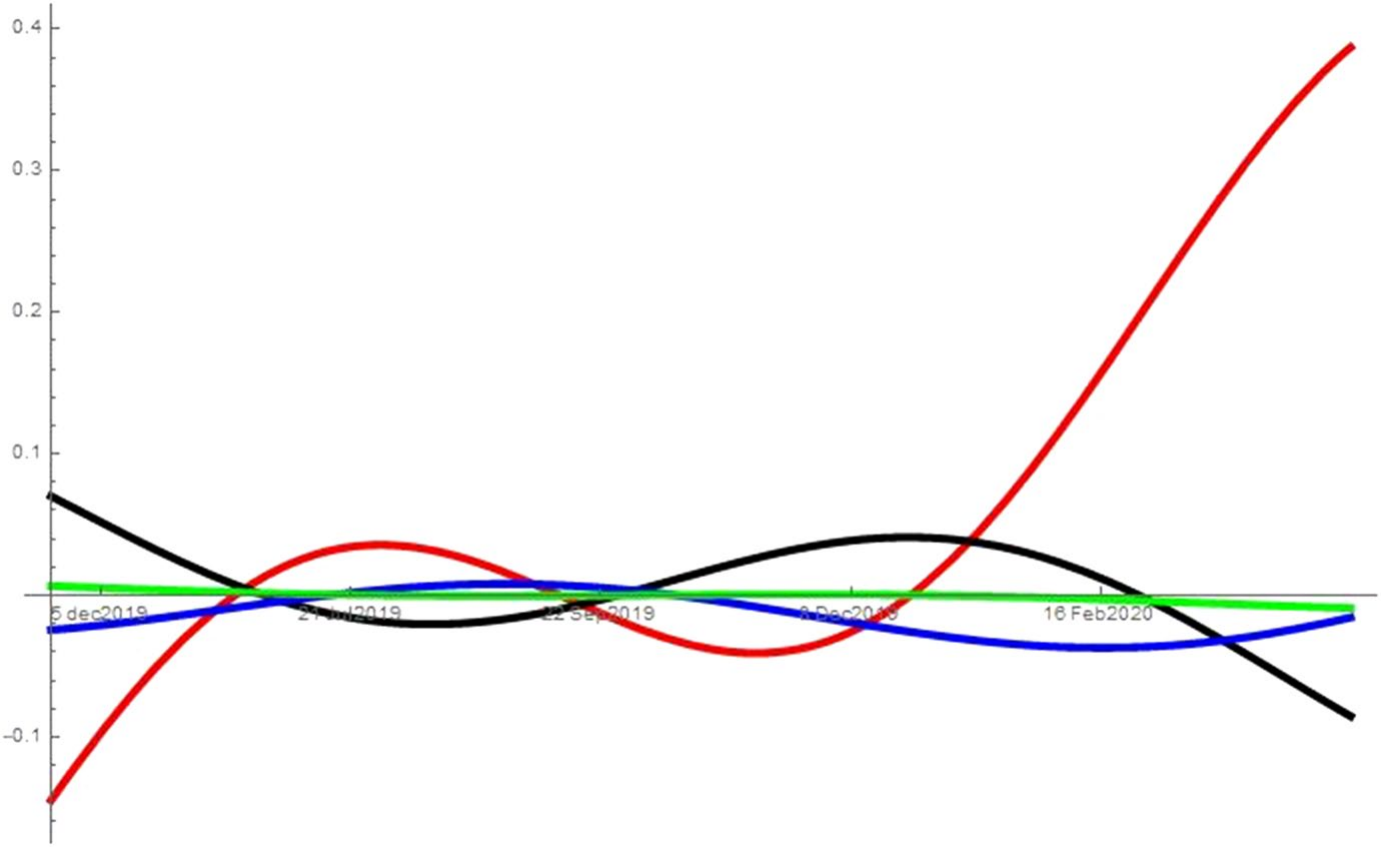
This figure shows the interaction coefficients of unemployment (red), symptoms (black), porn (blue) and news (green) for Utah

As noted in Sect. 2, I run the analysis with different combinations of searches for unemployment, symptoms, news, porn and alcoholic drinks. While the analysis refers to the combination unemployment, symptoms, news and porn, it can be applied also to the other two combinations tested: (*i*) unemployment, symptoms and news, and (*ii*) unemployment, symptoms, news and alcoholic drinks. When considering alcoholic drinks instead of porn in (*ii*), the searches for alcoholic drinks behave like the searches for porn in the specification described. That is, the results are consistent across all combinations considered and excluding the mediators (porn or alcoholic drinks) from the analysis does not affect the type of interactions the remaining variables have, e.g. excluding from the analysis searches for porn still leaves searches for unemployment as the predator of all other topics from the beginning of the pandemic. Figures 2, 3, 4, 5 and 6 show the interaction coefficients between the four searches in each of the areas considered, with time on the horizontal axis and the value of the interaction coefficients on the vertical axis. The kind of interaction remains fairly stable—the intensity of such interactions fairly weak—prior to the pandemic. Before the spring of 2019, news and porn (alcoholic drinks) are almost in neutralism, whereas they are in amensalism with searches for symptoms and in commensalism with searches for unemployment. Unemployment and symptoms are in a predator–prey relationship but the intensity of the interaction is really low. The trend of weak interactions changes completely around the beginning of the pandemic, as the intensity of the interactions explodes. This indicates that prior to the pandemic the four aspects considered—health, economy, porn (alcoholic drinks) and news—were only loosely connected in internet users’ minds. However, they became deeply intertwined during the pandemic. Second, after the outbreak of COVID-19, unemployment starts predating all other variables. In other words, an increase in searches for unemployment during this period leads to a decrease in searches for all the other topics considered. At the same time, increases in the searches for symptoms, porn (alcoholic drinks) and news lead to an increases in the share of searches for unemployment. The reason behind this finding could be that the United States have a limited welfare state compared to other western countries, so being unemployed can have immediate and grave repercussions. This means that unemployment crises swallow all other issues, and thus most Americans turn their attention away from other issues and focus on the economic crisis. At the same time, more news consumption in a time of severe crisis might also increase searches for unemployment. As people become more aware of the dire situation in which the US economy is, they become more concerned that many Americans—or even themselves and people they know—might remain unemployed. In fact, as unemployment increases, fears over the ability to pay even just for food have led some Americans to skip some meals (New York Times 2020). A perhaps more unexpected finding reveals the unique nature of this crisis. Usually, unemployment tends to be associated with worsened health conditions (Paul and Moser 2009), and therefore presumably with more Google searches related to symptoms. In this case, however, unemployment predates those searches. A preliminary caveat is that we are observing macro-level data on searches and hence individual level explanations are affected by the ecological fallacy. However, with this in mind, a possible explanation for this finding could be that unemployed people spend less time outside of their home, and therefore have a lower risk of contracting COVID-19. Thus, paradoxically, at least in the short-term unemployment has a positive impact on health during this pandemic. This would be reflected in a lower number of searches for symptoms. The pandemic can also help explaining the other side of the coin, namely why health is a prey of unemployment: when people are more worried about symptoms they also become more worried about unemployment. Contracting the virus could result in loss of job or income, and hence negative symptoms also cause worry in terms of unemployment. Moreover, unemployed people might search more for unemployment benefits and which medical bills would be covered under Cares Act or unemployment benefits, both part of searches for “unemployment” on Google Trends. A similar hypothesis can be advanced to explain the predator–prey relation between unemployment and porn (alcoholic drinks). As people become more worried about the ability of millions of Americans, and possibly themselves or their family and friends, to maintain a semblance of normal life, the refuge offered by these searches (and consumption of porn or alcohol) becomes insufficient. Therefore, those seeking a momentary distraction might be reminded of their more urgent issues and start searching for unemployment again. This dynamic suggests that when searches for unemployment increase, search for porn and alcohol are predated. Other interactions are also worth discussing. During the pandemic, porn (alcoholic drinks), news and symptoms proceed in mutualism, so when people search more for one of these topics, they also search more for the others. Therefore, for instance, as people read more news they also make more searches about symptoms. This could be because reading more news make them more worried about the pandemic, and hence make more searches about symptoms. Evidence from micro-level studies supports this hypothesis. For instance, a recent study finds a high correlation between people’s worry about the health crises caused by the pandemic and the amount of news they consume (Romano 2020). Moreover, as people become increasingly interested in news they also search more for porn and alcoholic drinks, maybe as a way to escape the anxiety created by the negative news. However, this is merely a conjecture as this study does not use micro-level data, and therefore explanation at the individual level might be affected by the ecological fallacy. The external validity of these results is higher because the States analysed and DC implemented different sets of restrictions, have been hit by the virus with different intensity and started at different levels of unemployment. Moreover, their governors and mayor are at different ends of the political spectrum. Therefore, it seems that the type of interactions between these searches are general and are not driven by specific features of the areas considered.

## Conclusions

The dynamics governing people’s Google searches are far more interesting than one could expect. Many people are likely to approach searches in the same way, so that looking at the interactions among topics searched can inform policymakers about people’s main sources of interest and how they interact with each other. Doing so through Google Trends data offers policymakers (and researchers) several advantages. First, Google Trends offers the possibility to freely browse through (almost) real-time information on what people search for. Second, unlike in surveys, Google Trends data allows policymakers to rely on revealed preferences, rather than stated preferences of participants. For searches like porn and alcohol this has substantial advantages, as people may not declare how often they search for these topics in a survey or on social media. People may intentionally post or avoid posting some content over others out of appearance concerns, which would bias any analysis. Finally, the format of this data, combined with Lotka–Volterra models, allows policymakers to derive the interactions among the topics searched. This work shows that not only the type but also the intensity of relationships between different searches can change during trying times. Moreover, there are common trends also across areas with different characteristics, therefore the lessons that can be learned here are likely to have external validity. More specifically, I find that the COVID-19 pandemic changed the type and the intensity of the interactions among searches for unemployment, symptoms, news and porn (alcoholic drinks). As the pandemic develops unemployment searches start predating all others. This first finding can be explained by the increase in unemployment that the U.S. has experienced from the beginning of the epidemic, coupled with a limited welfare state and worries about insurance coverage. Notably, instead of increasing people’s interest in health, the effect of the pandemic is to reduce the searches for symptoms relative to those for unemployment. This could be due to the specific nature of this pandemic, forcing many of the unemployed Americans at home due to restrictions to movement, and hence making them less exposed to the virus. The other variables are in a mutualistic relationship. As searches for news increase, so do searches for symptoms and porn, and as searches for symptoms and porn increase, so do searches for news. I find the same interactions in all the areas considered, despite the different policies adopted in the different states and the different incidence of the virus. Future research could investigate whether searches for news, unemployment and symptoms interact differently in other countries. For instance, one could replicate the analysis in a country with a more extensive welfare state. Alternatively, one could extend the analysis to other common searches that are likely to be related. One of such avenues could be looking at the type of online shopping websites people are looking for to understand whether they are prioritising purchases of essential items.
